# The genes of the sulphoquinovose catabolism in *Escherichia coli* are also associated with a previously unknown pathway of lactose degradation

**DOI:** 10.1038/s41598-018-21534-3

**Published:** 2018-02-16

**Authors:** Anna Kaznadzey, Pavel Shelyakin, Evgeniya Belousova, Aleksandra Eremina, Uliana Shvyreva, Darya Bykova, Vera Emelianenko, Anastasiya Korosteleva, Maria Tutukina, Mikhail S. Gelfand

**Affiliations:** 1 A. A. Kharkevich Institute for Information Transmission Problems, RAS, Bolshoy Karetny per. 19, Moscow, 127051 Russia; 2 N. I. Vavilov Institute of General Genetics, RAS, ul. Gubkina 3, Moscow, 119991 Russia; 3 M. V. Lomonosov Moscow State University, Vorobievy Gory 1-73, Moscow, 119991 Russia; 4 The University of Edinburgh, Alexander Crum Brown Rd, Edinburgh, Scotland EH9 3FF UK; 5 Institute of Cell Biophysics, RAS, Institutskaya 3, Pushchino, 142290 Russia; 6 Novosibirsk State University, ul. Pirogova 2, Novosibirsk, 630090 Russia; 7 Center for Data-Intensive Biomedicine and Biotechnology, Skolkovo Institute of Science and Technology, Moscow, 143028 Russia; 8 Faculty of Computer Science, Higher School of Economics, Kochnovsky pr. 3, Moscow, 125319 Russia

## Abstract

Comparative genomics analysis of conserved gene cassettes demonstrated resemblance between a recently described cassette of genes involved in sulphoquinovose degradation in *Escherichia coli* K-12 MG1655 and a *Bacilli* cassette linked with lactose degradation. Six genes from both cassettes had similar functions related to carbohydrate metabolism, namely, hydrolase, aldolase, kinase, isomerase, transporter, and transcription factor. The *Escherichia coli* sulphoglycolysis cassette was thus predicted to be associated with lactose degradation. This prediction was confirmed experimentally: expression of genes coding for aldolase (*yihT*), isomerase (*yihS*), and kinase (*yihV*) was dramatically increased during growth on lactose. These genes were previously shown to be activated during growth on sulphoquinovose, so our observation may indicate multi-functional capabilities of the respective proteins. Transcription starts for *yihT*, *yihV* and *yihW* were mapped *in silico, in vitro* and *in vivo*. Out of three promoters for *yihT*, one was active only during growth on lactose. We further showed that switches in *yihT* transcription are controlled by YihW, a DeoR-family transcription factor in the *Escherichia coli* cassette. YihW acted as a carbon source-dependent dual regulator involved in sustaining the baseline growth in the absence of *lac-*operon, with function either complementary, or opposite to a global regulator of carbohydrate metabolism, cAMP-CRP.

## Introduction

Catabolism and synthesis of carbohydrate molecules are important segments of bacterial metabolism. Many bacterial species can assimilate a wide range of carbohydrate substrates, adjusting to environmental changes, and are able to quickly switch between pathways. With multiple energy sources in the environment, a bacterial cell goes through metabolic assessment and, in most cases, selects a specific preferred energy source, e.g. glucose^1,2^.

Genes encoding proteins responsible for carbohydrate transport, degradation, and synthesis are very diverse. According to the IMG database^3^, over a hundred thousand genes related to carbohydrate metabolism have been described for over six hundred genomes of various bacterial species. Moreover, some of these genes are known to encode proteins that have multiple functions and can be involved in different metabolic pathways. For example, thermophilic glycoside hydrolase CoGH1A from *Caldicellulosiruptor owensensis* has a high capability of saccharification, being able to hydrolyze various substrates^4^, polysaccharide lyase Smlt1473 from *Stenotrophomonas maltophilia* is able to catalyze chemical bond cleavage in a variety of carbohydrate molecules^5^, and hexokinase Glk/TM1469 from *Thermotoga maritima* switches between substrates under different temperature conditions^6^.

Carbohydrate metabolism in most bacteria is controlled by the carbon catabolite repression (CCR) mechanism^7,8^. CCR allows the cell to select a preferred source over a non-preferred one, maintaining a proper energy balance^9^. In a glucose-free environment or under stress conditions bacteria switch to the next best carbon source available^10,11^. In *Escherichia coli* and related species, this switch is often controlled by cAMP-CRP as a global regulator, and a local regulator encoded by a gene usually located either in the same operon with genes encoding enzymes and/or transporters necessary for growth on alternative carbohydrates, or in a divergent orientation^12–14^.

cAMP-CRP is one of the most important transcription factors that regulates transcription initiation for more than a hundred genes of carbohydrate catabolism^15,16^. It functions as a dimer in the form of the CRP-cAMP complex and may activate or repress transcription. In most cases, CRP downregulates transcription when its operator region is situated upstream of the transcription start site^17,18^. CRP-mediated activation occurs when cAMP-CRP binds upstream of the target promoter and directly contacts RNA polymerase^19^. It can also bind DNA in a cAMP-independent manner, resulting in a weak target repression^17^. An additional, substrate-specific layer of regulation is provided by the binding sites of local transcription factors from six major families (LacI, ROK, DeoR, AraC, GntR, and TetR) that are located close to the CRP binding sites, or even overlap them. In most cases, these factors play a role opposite to CRP and act as dimers. Dimerisation caused by the ligand binding leads to a metabolic switch^20^.

Here, we identify and explore the second lactose catabolism system in *E. coli*, supplementing the *lac* operon described in the pioneer work of François Jacob and Jacques Monod^21^. The *lac* operon consists of three genes, encoding β-galactosidase, β-galactoside transacetylase, and β-galactoside permease. Until now, no other pathways for lactose utilisation in *E. coli* have been known.

In a recent biochemical study, Denger *et al*.^22^ described the *E. coli* gene cassette consisting of 10 *yih* genes and, according to that study, completing the biogeochemical cycle of sulphur. Four reactions of sulphoquinovose (SQ) catabolism were demonstrated using purified, heterologously expressed enzymes; other genes from this cassette were predicted to be involved in regulation, transport, hydrolysis, and other processes of sulphoglycolysis. The *yih* genes have not been linked to the lactose catabolism, and their transcription profile has not been characterised.

Unknown functions of a gene can be predicted using comparative genomics analysis, based on gene proximity in the chromosome and functional similarity of their protein products^23–29^. We compared functional composition of the *yih* cassette with other carbohydrate metabolism gene cassettes and found a match with a cassette involved in the lactose catabolism of *Bacilli*. Six genes from these two cassettes encoded proteins with similar functions, namely, hydrolase, aldolase, kinase, isomerase, transporter, and transcription factor, allowing us to suggest involvement of the *E. coli yih-*cassette in the lactose catabolism. We then confirmed it experimentally using expression analysis. We also mapped promoters for the *yih* genes, and studied how the switch to lactose utilization is regulated, thus describing a possible additional pathway for lactose utilization in *E. coli* and suggesting multi-functional features for the respective proteins.

## Results

### The functions of genes in the *E. coli* cassette encoding enzymes of sulfoquinovose degradation are similar to those in the lactose cassette in *Bacilli*

**Figure 1:**
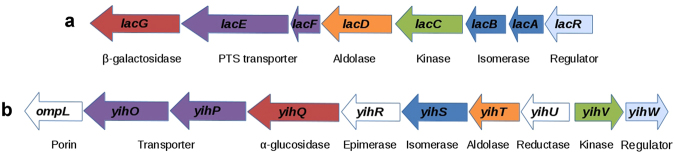
The lactose catabolism cassette of *Bacilli* (**a**) and the *yih* cassette of *Enterobacteriaceae* (**b**). Genes encoding proteins with similar functions are marked with the same colour. Genes marked white have no matches between the cassettes.

Comparison of the combinations of gene functions in cassettes related to bacterial carbohydrate metabolism revealed a six-gene match between a cassette linked with SQ degradation in *E. coli* and present in some other *Enterobacteriaceae*, and a lactose catabolism cassette in several *Bacilli* species (Fig. 1). Namely, the functional content of the *yih* cassette in a number of strains of *E. coli*, *Enterobacter cloacae*, *Salmonella enterica*, *Cronobacter turicensis*, and *Pantoea anantis*, all belonging to *Enterobacteriaceae*, matched the content in the lactose degradation cassette of the *Streptococcus* spp. (*S. gallolyticus*, *S. suis*, *S. pyogenes*, *S. agalactiae*, *S. uberis*, *S. equi*, *S. mutans*, and *S. sanguinis)* and *Staphylococcus* spp. (*S. aureus*, *S. epidermidis*, *S. haemolyticus*, and *S. lugdunensis*).

**Figure 2:**
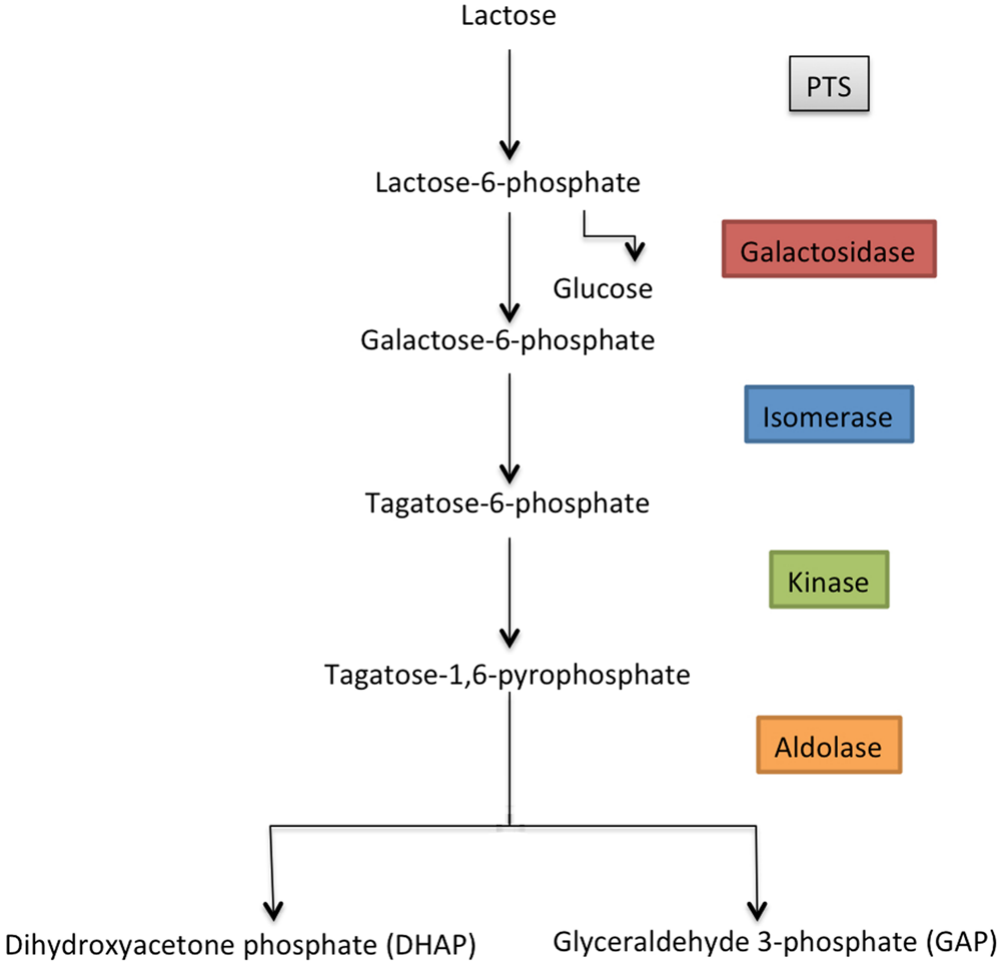
The lactose catabolism pathway encoded by the *Bacilli* cassette.

These matching genes encoded enzymes converting sugar substrates which shared at least three first numbers of the Enzyme Commission nomenclature, thus having similar chemical reaction types catalysed, namely hydrolases (3.2.1), aldolases (EC 4.1.2), kinases (EC 2.7.1), and isomerases (2.3.1), and also transporters and transcription factors (Fig. 2)^3^. The genes coding for aldolases also belonged to one and the same cluster of orthologous genes (COG3684). Such co-localization of genes linked with functions similar to those required for the *Bacilli* lactose pathway (Fig. 2) in the *Enterobacteriaceae* cassette allowed us to predict lactose degrading capabilities of the latter.

Our next goal was to test whether this *E. coli* cassette is actually involved in the lactose metabolism. Since no experimentally confirmed data on positioning of transcription start points for the genes of the *yih* cassette were available, the first step was to map promoters for the main genes encoding the key enzymes of the suggested catabolic pathway.

### Promoter mapping

**Figure 3:**
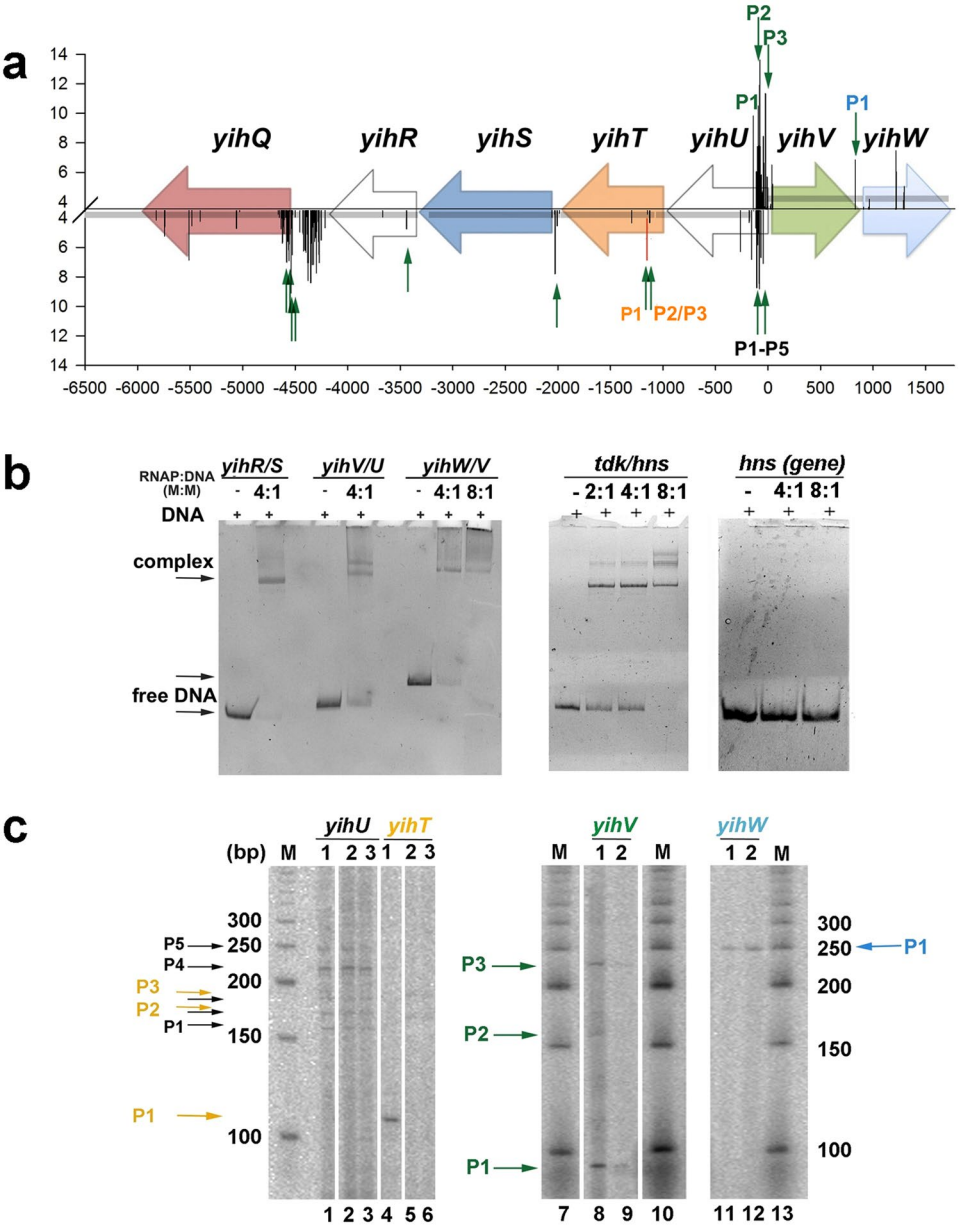
Promoter mapping within the *yih* cassette. (**a**) Schematic representation of the *yih* genes and candidate promoters predicted *in silico*. Horizontal arrows show gene positioning. Bars represent the transcription start points predicted by PlatProm on both DNA strands, their scores are indicated on the Y axis. Score assigned to the yihTP1 promoter by the unified algorithm, PlatPromU, is marked with red. X axis represents positioning related to the *yihV* ATG start codon. Green vertical arrows point out the 5′-end of RNA detected by the 5′-end-specific RNA-Seq^31^. (**b**) Band-shift assays with σ^70^-RNA polymerase suggests the presence of single promoters in the intergenic regions *yihV/W* and *yihS/R*, and two promoters in *yihU/V*. Molar ratios are indicated above the lanes. All samples were run on one gel. Known σ^70^-promoter of the *hns* gene was used as a positive control; intergenic region within *hns* where no promoters were predicted was used as a negative control (**c**) Primer extension analysis revealed transcription start point for *yihW* and several starts for the *yihU*, *yihT*, and *yihV* genes, some of which were activated during growth on lactose. (1) Growth on lactose, (2) growth on glucose, (3) growth on glycerol. The *yihU* and *yihT* samples, as well as the DNA ladder on the left, were run on one gel, while *yihV* and *yihW* were run on another gel, together with the DNA ladders displayed. Lanes that were taken from different parts of the gels are divided by spacing.

Computational analysis of the intergenic regions by phylogenetic footprinting and promoter search algorithm PlatProm^30^ allowed us to predict candidate promoters for the genes encoding aldolase (*yihT)*, kinase (*yihV*), isomerase (*yihS*), alpha-glucosidase (*yihQ*), and a local transcription factor (*yihW*). To obtain a comprehensive view on the transcription pattern in this locus, the *yihU* and *yihQ* genes coding for aldolase and aldose epimerase that are not associated with the suggested pathway, were also incorporated in the analyses (Fig. 3a). Computational predictions were then compared with the starts detected in 5′-end specific RNA-seq^31^ (Fig. 3a), and transcription starts for the mRNAs of the key enzymes were further confirmed experimentally using band-shift assays (Fig. 3b) and primer extension (Fig. 3c). Band-shift assays with σ^70^-RNA polymerase (RNAP) revealed the presence of promoters capable of efficient binding with RNAP in the intergenic regions *yihU/V*, *yihV/W*, and *yihS/R* (Fig. 3b). Formation of two complexes with almost identical efficiency suggested the presence of at least two equally strong promoters between *yihU* and *yihV*. Specificity of the RNAP binding was confirmed by its strong interaction with known σ^70^-promoter of the *hns* gene and the absence of complex formation with the intergenic region of *hns* where no promoters had been mapped (Fig. 3b). To map the exact transcription start points and to test whether these promoters are lactose-sensitive, primer extension was done with RNA isolated from cells growing for 6 hours on M9 + 10% LB supplemented with 0.2% lactose, glucose, or glycerol. Reverse transcription with primer yihU_RT revealed several products (black arrows in Fig. 3c) corresponding to the transcription starts at positions −15 (PlatProm score 6.83), −25 (5.04), −35 (3.52), −62/−63 (8.75), −82/−92 (a large cluster with scores 8.75–13.61, *yihU* promoters yihUP1-yihUP5) relative to the annotated ATG codon. RNA synthesis from yihUP1, yihUP2, yihUP4, and yihUP5 was also confirmed with primer yihU/yihV_F (data not shown, Supplementary Table 1) and 5′-end specific RNA-seq^31^ (green arrows in Fig. 3a). Note, however, that none of these promoters was dependent on a carbon source suggesting that the protein product of *yihU* (putative reductase) is not involved in the lactose metabolism. For *yihV* (putative kinase), several transcription starts were also mapped, with the major one located at –25/–27 relative to the starting ATG (a large cluster of predicted promoters with the highest score of 11.35 at position 4071737 in U00096.2). This promoter was activated during growth on lactose (Fig. 3c). For *yihT*, three possible starts were detected (orange arrows in Fig. 3c). Two of them, corresponding to yihTP2 and P3, located +35/+45 relative to the putative ATG codon, were predicted with a relatively low score of 3.67–4.33 and, in line with this, had low transcriptional activity. Nonetheless, these products were not detected during cell growth on lactose (lane 1 in Fig. 3c), while another promoter (yihTP1) was switched on, with transcription start located at +93/+94 relative to ATG (lane 4 in Fig. 3c).

This promoter was predicted only with the universal algorithm, PlatPromU, suggesting its recognition by an alternative sigma factor. The existence of transcriptional switch is also supported by the stringent control discriminator GCGC motif present between the transcription start and the −10 element of yihTP1. All mapped promoters for *yihT* are located within the proposed ORF for this gene suggesting either incorrect identification of the translational start (at least four additional candidate start-codons were detected, 51, 78, 84 and 87 nt downstream), or their regulatory role in the *yihT* transcription^32^. For *yihW*, only one promoter was mapped, with transcription start located at −27/−28 relative to the ATG codon (Fig. 3a–c).

### Expression of the *yih* genes during growth on various carbon sources

**Figure 4:**
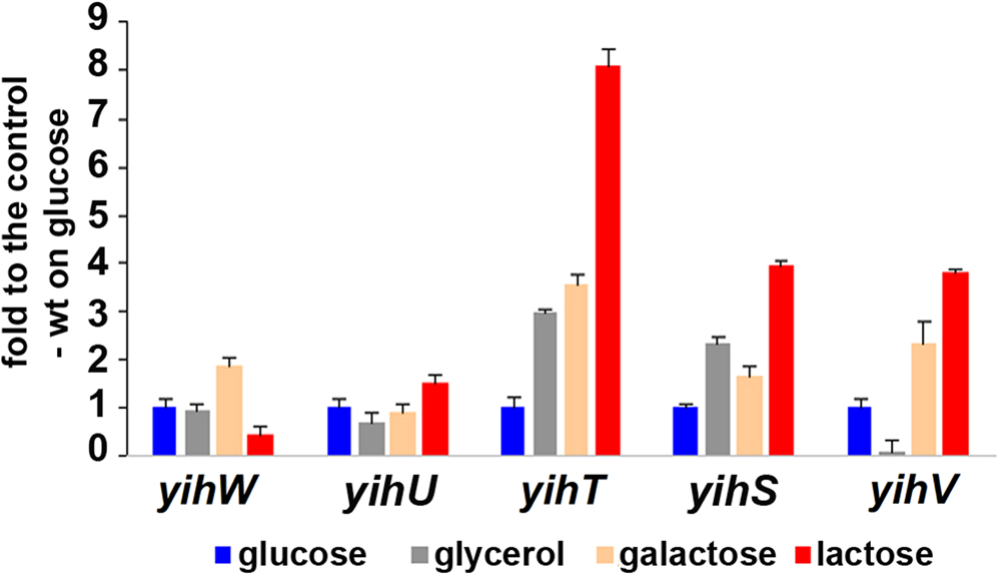
mRNA levels of the *yih* genes measured by qRT-PCR. Growth conditions are indicated at the bottom. No changes were detected in the *hns*-mRNA and *ysaA*-aRNA levels that were used as controls. mRNA levels are expressed relative to their levels during growth on glucose. Standard deviations were calculated on the basis of three biological and three technical replicates.

To further check whether transcription of the *yih* genes is lactose-dependent, we tested their mRNA levels in cultures growing in the same conditions as used for primer extension, and also during growth on galactose. Glucose was taken as a standard rich carbon source, glycerol as a standard poor carbon source, and galactose as an intermediate of the possible pathway shown in Fig. 2. Results of qRT-PCR tests for cultures grown on various carbon sources for 6 hours are shown in Fig. 4. Genes encoding kinase (*yihV*), aldolase (*yihT*), and isomerase (*yihS*) were activated during growth on galactose and lactose, and this activation was more pronounced during growth on lactose. *yihW* expression, conversely, was approximately 2-fold repressed during growth with lactose suggesting either its function as a repressor for the *yih* genes at this time point, or a self-repressor function. The *yihU* gene, encoding a putative reductase, did not respond to differences in carbon sources (Fig. 4).

Expression of *yihR* and *yihQ* (data not shown), encoding epimerase and glucosidase, respectively, was also independent (*yihR*) or barely dependent (*yihQ*) on the carbohydrates used; this result supports our previous assumption about their independent transcription (Fig. 3) and suggests that these genes are not related to the lactose metabolism. Comparative analysis showed that a number of *Enterobacteriaceae* bacteria, including *Escherichia albertii* and *Citrobacter koseri* strains, have orthologs only for the *yihSTUVW* genes, while the rest of this cassette, including *yihR* and *yihQ*, is missing, which also indicates that different parts of the 10-gene cassette may function separately.

### cAMP-CRP and YihW are involved in the regulation of the *yih* genes

A moderate decrease of the *yihW*-mRNA level in the presence of lactose suggested that the YihW transcription factor may be involved in down-regulation of the *yih* genes as a local regulator. If so, it may be complemented by cAMP-CRP as a global regulator. Thus, we searched for potential CRP binding sites near the mapped promoters and tested whether these promoters are CRP-dependent.

**Figure 5:**
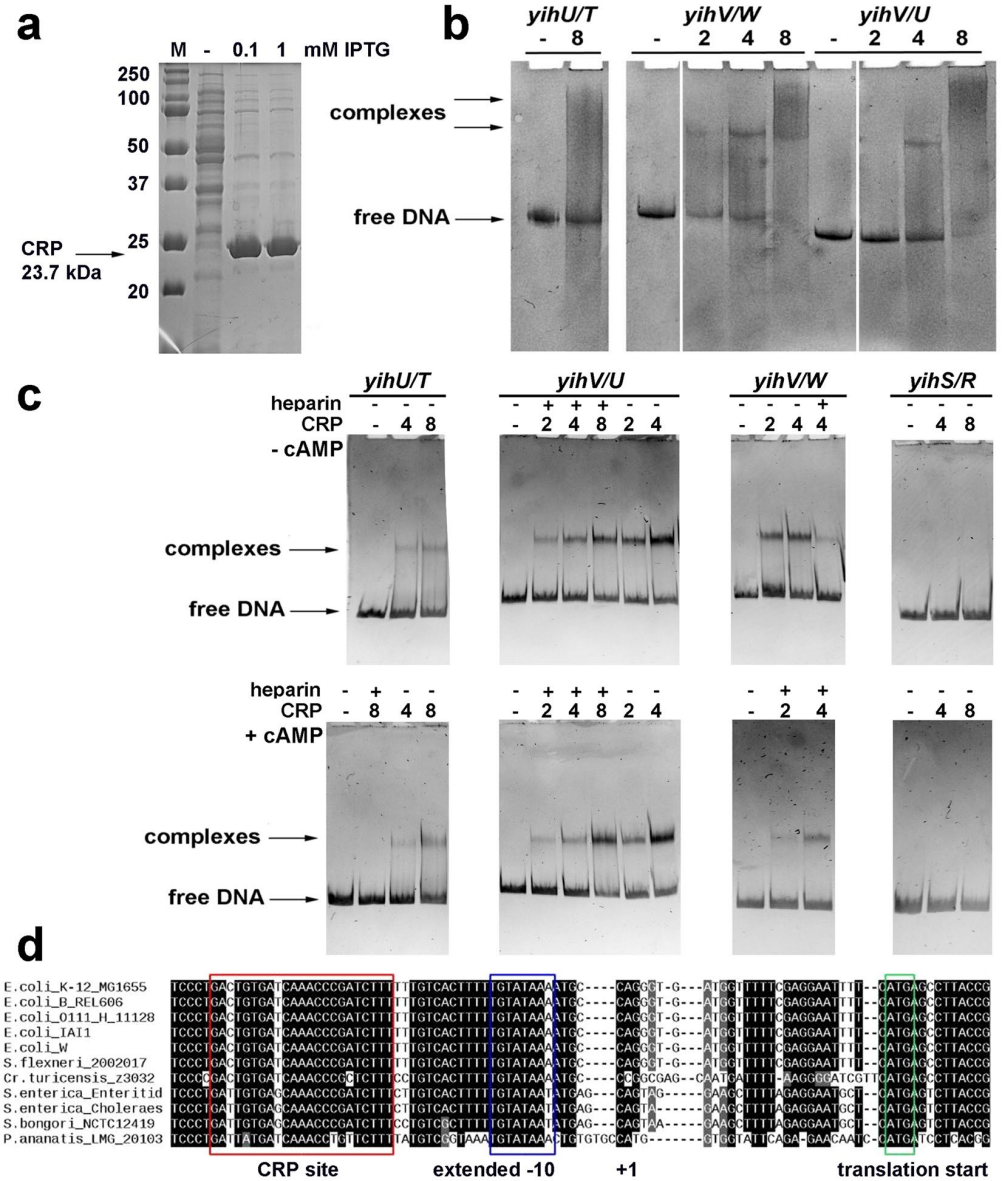
(**a**) The level of cAMP-CRP synthesized in the cell from pET_CRP after IPTG induction. (**b**) cAMP-CRP interacted with the *yihU/yihT*, *yihV/yihW*, and *yihV/yihU* intergenic regions, with most efficient binding being registered for *yihV/W*. The protein:DNA ratio is indicated above the lanes. Control lane for the *yihV/yihW* intergenic region was run on a separate gel, as well as the sample with 1:1 protein:DNA ratio (this ratio gave no relevant binding and the corresponding lanes were deleted from the final figure). (**c**) Binding of purified CRP to the studied regions with/without cAMP. The protein:DNA ratio and the presence of heparin in the sample is indicated above the lanes. (**d**) Multiple alignment of the *yihV/W* intergenic region in several bacteria containing orthologs for these genes revealed high conservation of the mapped *yihW* promoter and the CRP binding motifs.

The sites were found in the *yihT/U*, *yihU/V*, and *yihV/W* intergenic regions and were further confirmed by phylogenetic footprinting and band-shifts assays with native CRP from the cell lysate (Fig. 5b; CRP expression level is shown in Fig. 5a). The presence of CRP in the formed complexes was confirmed by Western blot analysis. Strong specific binding of CRP to the *yihV/yihU* and *yihW* promoters was also detected with the purified protein, and interaction with both promoter regions was ~30% enhanced in the presence of cAMP (Fig. 5c). In line with this, a highly conserved site was found in the *yihV/W* intergenic region at the position −41.5 relative to the start site of the *yihW* promoter, that is typical for the Class II CRP-dependent promoters (Fig. 5d).

Assuming that CRP acts as a global regulator for all *yih* genes, and YihW is their local regulator, we investigated the growth rates of K-12 MG1655 cells with deleted *yihW* and *crp*^33^ on glucose and lactose.

**Figure 6:**
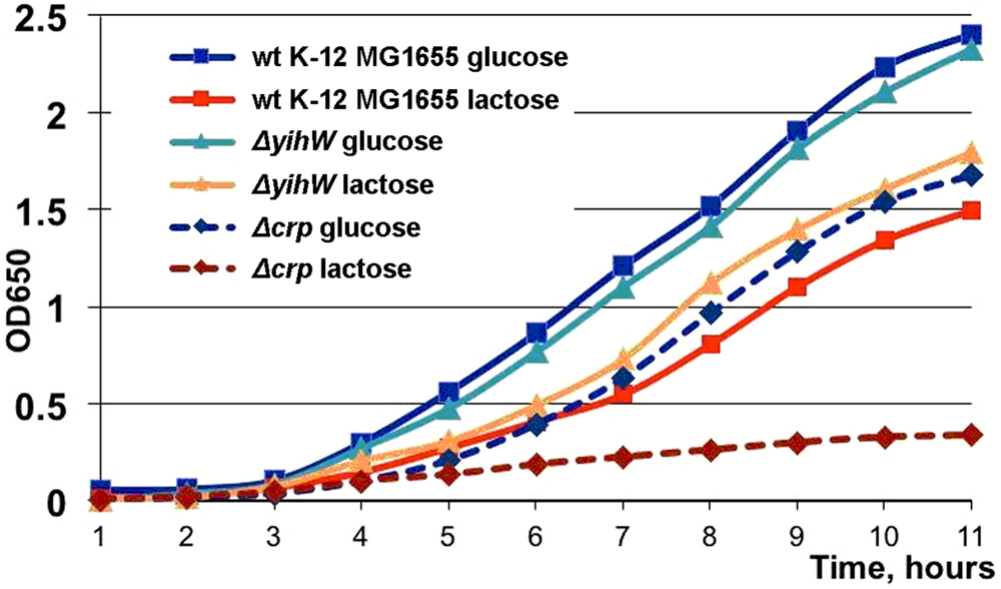
Effects of *yihW* and *crp* deletions on growth in the presence of 0.2% glucose or lactose. Squares: the parent strain; triangles: the *yihW* mutant. Dashed lines correspond to the cultures with deleted *crp*. Each growth curve is an average based on three independent measurements.

The wild type and the *yihW* deletion strains grew equally well in the presence of glucose, with slightly retarded growth of *Δcrp* reflecting the key role of CRP in the regulation of glucose metabolism. The parent strain grew more slowly on lactose than on glucose as the carbon source, but deletion of *yihW* resulted in more rapid growth on lactose (Fig. 6). In contrast, growth of the *crp* mutant on lactose almost stopped as soon as the LB had been consumed. These results reveal that YihW is likely involved in regulation of the lactose metabolism and may play the role opposite to CRP. The next aim, therefore, was to uncover the roles of these proteins in the transcription of the *yih* genes.

### Effects of cAMP-CRP and YihW on transcription of the *yih* genes

**Figure 7:**
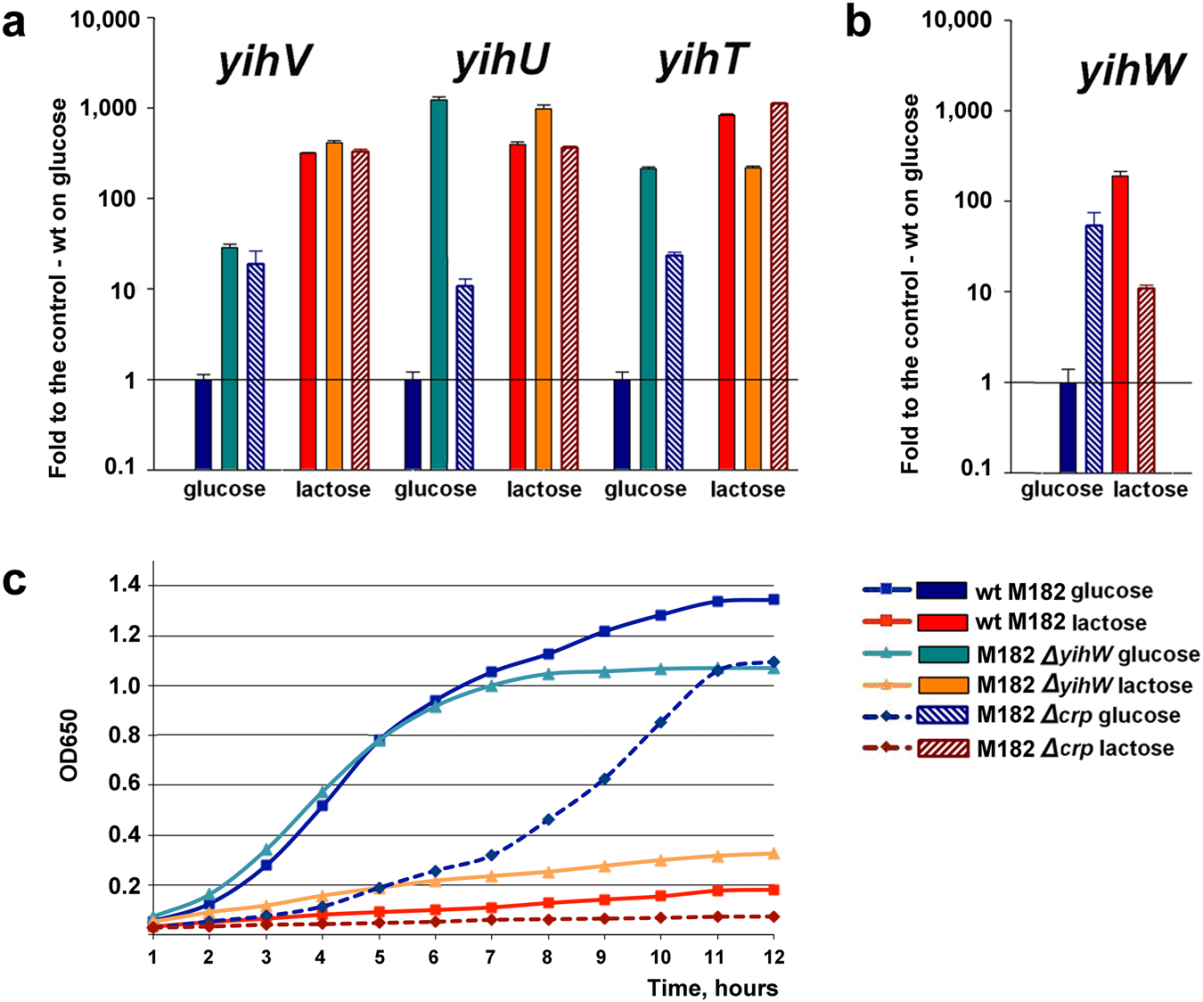
Effects of *yihW* and *crp* deletion on mRNA levels the *yih* genes (**a**,**b**), and culture growth on glucose or lactose (**c**). Growth conditions are indicated at the bottom right. The mRNA levels are expressed relative to their levels in the parent strain during growth on glucose. No changes were detected in the *hns*-mRNA and *ysaA*-aRNA levels that were used as controls. Standard deviations were calculated on the basis of three biological and three technical replicates. Growth curve in (**c**) is an average based on three independent measurements. Squares: the parent strain; triangles: the *yihW* mutant. Dashed lines correspond to cultures with deleted *crp*.

Transcription of the *yihT*, *yihS*, and *yihV* genes was activated after 6 hours of growth on lactose (Fig. 4), while *yihW* was slightly repressed. Together with affected growth (Fig. 6), this suggested involvement of YihW in the control of the *yih* genes that was confirmed with qRT-PCR (Fig. 7). At that, RNA was isolated from the *lac*-deficient *E. coli* M182 cells^34,35^ exponentially growing in the minimal medium containing twice less LB (5%) to get more pronounced effects of the carbon source. The experiment showed that *yihT* expression on both glucose and lactose is totally controlled by YihW that functions as a sugar-dependent dual regulator (Fig. 7a), and repressed by CRP during growth on glucose. Expression of *yihW* itself is up-regulated by CRP in the presence of lactose, and down-regulated on glucose (Fig. 7b). Finally, both YihW and CRP function as repressors for *yihV* (Fig. 7a).

## Discussion

Since Jacob and Monod described the *lac* operon in late 1950s^21^, no other pathway for lactose utilisation has been observed in *Escherichia coli*. Here, we demonstrate that the *yih* cassette of *E. coli*, previously linked with SQ degradation^22^, may be also involved in lactose catabolism, thus suggesting the existence of an alternative pathway of further lactose degradation following initial hydrolysis. This hypothesis was initially stated based on the comparative genomics analysis, and then confirmed in a number of experiments.

The lactose catabolism pathway of some *Bacilli* species, such as *Streptococcus*, *Staphylococcus*, and *Lactococcus* spp., differs from the common lactose degrading pathway, which involves initial hydrolytic cleavage of lactose to glucose and galactose by β-galactosidase. The *Bacilli* species do not hydrolyse lactose directly; they transport it into the cell by a phosphoenolpyruvate-dependent phosphotransferase system that phosphorylates lactose to lactose-6-phosphate^36^. Then, phospho-β-galactosidase hydrolytically cleaves the phosphorylated derivative of lactose into glucose and galactose-6-phosphate^37,38^. Finally, D-galactose-6-phosphate is degraded via the D-tagatose-6-phosphate pathway comprised of D-galactose-6-phosphate isomerase^38^, D-tagatose-6-phosphate kinase^39^, and D-tagatose-1,6-diphosphate aldolase^40^ (Fig. 1).

Based on the concordance between functions encoded in the *yih* cassette and the *Bacilli* cassette, which is linked with the lactose catabolism, we suggested that the proteins encoded by the *yih* cassette could be involved in a similar lactose degradation pathway. To validate this prediction, we performed a detailed analysis of gene expression in different growth conditions. As one of quantitative characteristics indicating involvement of a particular enzyme in conversion of a particular substrate is changing expression of its gene in response to this substrate, we first compared expression of genes encoding the key enzymes of the predicted pathway during growth on glucose, galactose, and lactose. Following initial lactose hydrolysis, it should further be degraded by kinase (YihV), aldolase (YihT), and isomerase (YihS) (Figs 1 and 2). Initial hydrolysis is possibly performed by β-galactosidase from the *lac*-operon; however, even in the absence of *lac-*operon, all strains, except *∆crp*, were capable of slow growth with lactose and galactose, thus suggesting possible existence of an alternative way of lactose cleavage (Fig. 7, Supplementary Figs S2 and S3).

Indeed, qRT-PCR demonstrated that all three respective genes were substantially activated on both lactose and galactose, the next intermediate of the pathway. As shown in Figs 4 and 7, these genes were activated after either four or six hours of growth. Note, however, that the *yihW* gene, encoding a predicted DeoR-family transcriptional regulator, was also lactose-activated during exponential growth but repressed upon transition to starvation where the substrates start depleting. This observation led us to an assumption that YihW may act as a dual regulator for the *yih* genes, activating them in the presence of extra lactose and repressing when lactose is lacking. Stimulated growth of *lac*-minus cultures with lactose in the absence of YihW, that is even more pronounced on galactose (Fig. 7 and Supplementary Fig. S3), is of particular interest. This observation might support involvement of YihW and the whole *yih* cassette in sustaining the baseline *E. coli* growth with lactose in the absence of *lac-*operon.

Effects of local regulators like YihW are usually opposed or supported, depending on growth conditions, by the action of global regulators; in the case of sugar metabolism of *E. coli*, that is cAMP-CRP^15,19^. Figures 6 and 7 clearly show that YihW may play a role opposite to that of CRP.

**Figure 8:**
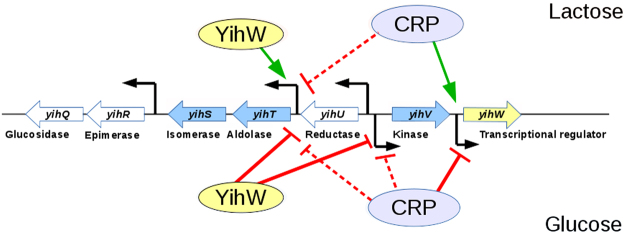
Regulation of the studied *yih* genes during growth on lactose (top) and glucose (bottom) by the CRP and YihW transcription factors. Green arrows indicate activation of transcription, red lines indicate repression. Dashed lines indicate cases where inhibition is moderate and may be due to indirect effects.

To further check this assumption and to reconstruct regulatory events that take place in the *yih* locus upon switching the carbon source from glucose to lactose and *vice versa*, we examined how expression of the key genes would change in the absence of CRP and YihW. The results are shown in Fig. 7a,b and summarized in Fig. 8. Activation of *yihW* itself during exponential growth with lactose was CRP-dependent, due to direct binding of CRP at −41.5 relative to the *yihW* promoter mapped here (Figs 3c and 5b). When lactose was absent, CRP repressed the *yihW* expression.

YihW, in turn, is critical for the balanced expression of the aldolase gene *yihT*; it is responsible for the lactose-driven activation and for tight repression in the absence of lactose. Most probably, the same is also true for the isomerase gene *yihS*, because its transcription profile is almost identical to that of *yihT* (Fig. 4). When *yihW* was deleted, the *yihT* expression became totally independent on the carbon source (Fig. 7). CRP here plays a role of sugar-independent repressor due to its weak binding in a cAMP-independent manner^16^. This was consistent with positioning of its binding sites found by phylogenetic footprinting^17^ and was further confirmed by band-shift assays for the *yihT/U* intergenic region (Fig. 5b,c).

Interestingly, YihT predicted to be 6-deoxy-6-sulphofructose-1-phosphate aldolase is homologous to tagatose 1,6-diphosphate aldolase LacD present both in various *Salmonella* and *Shigella* species, and in several *E. coli* strains such as *E. coli* APEC O1 and *E. coli* O157:H7. The nucleotide sequence identity is 80% or even higher, with most substitutions occurring in the third codon positions, yielding 96% conservation of the respective amino acid sequences. Organization of the genomic neighborhood of these genes in *Enterobacteriaceae* strains differs, but in most cases *yihT* and *lacD are* accompanied by divergently transcribed genes homologous to *yihW*.

The *yihV* gene expression is also activated during growth on lactose and repressed on glucose, controlled by both YihW and CRP (Fig. 8).

This case is an example of a successful prediction of gene functions based on their co-localization patterns. The revealed complexity of the *yih* locus in *E. coli* seems to represent a case where, depending on environmental conditions, bacteria use the same set of multifunctional enzymes in different pathways, exploiting a finely-tuned regulatory mechanism.

Demonstrating possible involvement of the *yih* genes, previously known for only a very narrow specialization in SQ degradation, in lactose metabolism, our study raises a number of further questions concerning biochemical specialization of the proteins encoded by the *yih* genes, particularly, their specificity and affinity to lactose and SQ, and the regulation of their expression during growth on SQ.

## Materials and Methods

### Cassette database and comparison

We previously compiled a database^41^ comprising over a hundred and forty-five thousand genes linked to carbohydrate metabolism according to the IMG database^3^ from over six hundred bacterial species. Gene functions were identified based on the Enzyme Nomenclature numbers provided in the IMG annotation. Cassettes were assembled based on co-localization of these genes in bacterial chromosomes. Genes were assigned to the same cassette if a distance between adjacent genes was less than 300 bp; the order of genes and strands were not taken into consideration. The database contains over twenty-six thousand cassettes. Tools developed *ad hoc* were used to search the database for conserved function combinations within cassettes belonging to different species.

### Strains, plasmids and growth conditions

All strains and plasmids used in this study are listed in Supplementary Table S1 online. The *yihW* and *crp* genes were disrupted in *E. coli* BW25113 using recombineering^42^. The *gene::kan* mutations were then transferred by P1-transduction into *E. coli* K-12 MG1655^43^ or M182, which is a *lac*-minus derivative of K-12^34^. Cells were grown in the minimal salts (MS) medium supplemented with 5% or 10% (v/v) LB, 0.2% (w/v) of the appropriate carbon source - D-glucose, D-galactose, lactose or glycerol. Cultures were grown aerobically at 37 °C under constant shaking. Cells were harvested after 4.5 hours of growth (mid-log phase with OD~0.2–0.4, depending on the strain). To express the cAMP-CRP protein, *E. coli* BL21* (DE3) cells^44^ were chemically transformed with the pET_CRP plasmid constructed based on pET28b (Invitrogen), with the *crp* gene inserted between the NdeI and Bpu1102 sites. Purified transformants were grown on LB or Terrific broth (TB) at 37 °C till OD_650_ = 0.3, and the protein synthesis was induced by addition of a very low IPTG concentration (20 μM) to avoid the effects of potential protein toxicity. The induction rate of CRP after 3 hours of IPTG induction was ~70% of the total protein (See Supplementary Fig. S1).

### CRP purification

To obtain pure CRP, BL21*(DE3) cells with overproduced CRP (16 hours of induction with 20 μM IPTG in 200 ml TB, Amp100) were harvested by centrifugation at +4 °C, washed with 1xPBS and lysed with 2 ml BugBuster protein extraction reagent (Novagen) for 20 minutes at +4 °C. Due to the very high induction level, all protein was in insoluble fraction even when very low IPTG concentration and low temperatures were used. To extract CRP from inclusion bodies, two-step lysis was performed: after initial lysis with BugBuster, 6 ml of ice-cold Lysis buffer^15,19^ (50 mM KPO_4_ buffer pH 7.5, 2 mM EDTA, 0.2 M NaCl, 5% w/v glycerol, 2 mM DTT, 50 μg/ml PMSF) was added, and cells were further sonicated 3 × 30 seconds on ice. Then, the lysate was cleared by 20 min centrifugation at 15,000 rpm, +4 °C, and loaded onto cAMP-agarose (Sigma). Then, the protocol described in^15,19^ was applied. Final yield of purified native CRP was ~20 mg of protein for 200 ml culture. All steps of purification are illustrated in Supplementary Fig. S1.

### Promoter mapping

Four intergenic regions with lengths allowing for promoter or transcription factor site mapping, e.g., exceeding 40–50 nucleotides, were selected for genes of the *yih* cassettes from the *Escherichia coli*, *Enterobacter cloacae*, *Salmonella enterica*, *Cronobacter turicensis*, and *Pantoea anantis* (namely, the *yih*R/S, *yih*T/U, *yih*U/V, and *yih*V/W regions). Regions were then extended in both directions by 100 nucleotides allowing for possible errors in the original open reading frame assignments. The resulting nucleotide sequences were obtained from GenBank^45^ and aligned using the T-Coffee multiple alignment tool^46^. Promoter-like motifs in the *E. coli* K-12 MG1655 genome (U00096.2) were found by the software PlatProm^30^ and its unified version PlatPromU^47^ where the impact of a sigma factor in promoter recognition by RNAP was depleted. Transcription starts were localized by band-shift assays with σ^70^-RNA polymerase, single-round transcription *in vitro* as previously described^48^ and by measuring the lengths of primer extension products *in vivo*. For primer extension, cells were grown in MS in the presence of 10% LB and 0.2% glucose, lactose, or glycerol, and harvested at OD_650_ = 0.4. RNA was extracted from 10 ml of cell culture with TRIzol reagent (Invitrogen, USA) and treated with DNAse I (New England Biolabs, USA) following the manufacturers’ instructions. 10 µg of total RNA was incubated with 4 pmol of the [γ-^32^P]ATP-labeled appropriate primer (Supplementary Table S2) and SuperScript II reverse transcriptase (Invitrogen, USA) according to the manufacturer’s protocol. The resulting samples were preheated and loaded onto a 6% denaturing polyacrylamide gel calibrated with [γ-^32^P]ATP-labeled 50 bp DNA ladder (New England Biolabs, USA). Gels were scanned using PMI Molecular Imager (Bio-Rad, USA) and autoradiographed.

### Search for transcription factor binding sites

Candidate binding sites for transcription factors were identified in the intergenic regions using phylogenetic footprinting and the Virtual Footprint database^49^, with respect to the mapped transcription start sites, within the region −250/+50. The resulting sites were ranked according to their scores and conservation among the *Enterobacteriaceae* species. Highly conserved sites with best scores were retained for further analysis.

### Electrophoretic mobility shift assays

Electrophoretic mobility shift assays (EMSA, band-shift assays) were used to test the efficiency of the RNA polymerase binding to the regulatory regions of the genes belonging to the *yih* cassette. DNA fragments containing potential promoter regions of separate genes were PCR amplified (primers marked as R and F for each gene, Supplementary Table S2), extracted from the gel and used for subsequent experiments. One pmol of DNA was incubated at 37 °C in 1× Transcription buffer^50^, for 10 min, then 1 to 4 pmol of purified σ^70-^RNA polymerase (obtained as described in ref.^50^) was added. After further 30 min at 37 °C, complexes were loaded onto a 5% polyacrylamide gel that had been pre-warmed to 37 °C. Protein complexes were separated from free DNA by electrophoresis in 1xTBE buffer. The DNA fragment containing known σ^70^-promoter of the *hns* gene amplified with primers hns_Bgl_263 and hns_Xba (Table S2) was used as a positive control; intergenic region within *hns* (primers RT-PCR) where no promoters were predicted was used as a negative control.

The same approach, but with cell lysate with the overproduced protein, was used to test the ability of cAMP-CRP to bind the regulatory regions of the *yih* genes. This approach allowed us to skip addition of extracellular cAMP in the sample *in vitro*. CRP was produced in BL21*(DE3) cells, then cells (10 ml) were harvested, washed and resuspended in 3 ml of 0.5× Transcription buffer with 10 mM PMSF. Cell lysate was prepared using standard 3 × 30 seconds rounds of sonication, and a total protein concentration was determined using the Bradford reagent (Sigma, USA). Approximate molar concentration of CRP in the lysate was calculated, and 1–8 molar excess of the protein was used in band-shifts.

To estimate the efficiency of binding, free DNA fragments and proteins were loaded on separate lanes. Gels were stained by ethidium bromide to reveal bands containing DNA. Western blotting was used for specific detection of CRP in the complexes. In brief, band-shifts were performed as above in duplicate, one of which was stained with ethidium bromide and the other one was transferred onto a PVDF membrane (Immobilon, Sigma-Aldrich, USA) using the Mini trans-blot protocol (Bio-rad, USA). The membrane was then blocked with 0.5% skimmed milk (Oxoid, UK) and incubated with anti-CRP (T14) antibodies (Cell Signalling, USA) in Tris-buffered saline (TBS) supplemented with 0.1% Tween-20 and 0.5% skimmed milk for 2 hours at 37 °C. After 3 × 10 min washes with TBS-T, secondary antibodies (A3687, anti-rabbit IgG, Sigma, USA) were added and incubation was allowed for 1 hour at room temperature. Membranes were stained with Western-blue stabilized substrate for alkaline phosphatase (Promega, USA) and scanned. To prove specific binding of CRP, band-shifts were also made with the purified protein with or without addition of cAMP. Band-shifts were made exactly as above, but 200 μM cAMP was added to the samples, to the gel and the running 1xTBE buffer^15^, and 20 μg/ml heparin was added to some samples to prove or disprove binding specificity.

### Quantitative PCR

DT-322 thermocycler (DNA-Technology, Russia) and SYBR Green I as a fluorescent dye (Invitrogen, USA) were used for quantitative PCR (qRT-PCR). Primers used for reverse transcription (−RT) and amplification (−PCR) are listed in Supplementary Table S2. No PCR products were detected in negative controls in the absence of reverse transcriptase. To avoid influence of the changed growth, both *hns* and *ysaA*-aRNA^51^ were used as controls. Data obtained from at least three biological samples and analyzed in three statistical replicates were calculated by the ΔΔC_t_ method. The error bars indicate the standard deviations of the respective mean values.

### Availability of data and material

Data generated or analyzed during this study are included in the present manuscript, additional datasets analyzed during the current study are available from the corresponding author by request. All strains and plasmids are also available by request.

### Software used for figure preparation

Schemes in Figs 1, 2 and 8 were prepared with Libre Office Draw and Multiple Align Show (http://www.bioinformatics.org/sms/multi_align.html). Graph in Fig. 3a was first built in SigmaPlot 8 and finalized in Adobe Photoshop CS5. Gels shown in Figs 3b, 5a–c were photographed, converted to a greyscale, and the text was added in Adobe Photoshop CS5. Images shown in Fig. 3c were taken from the PMI Molecular Imager, then resolution was increased and the text was added in Adobe Photoshop CS5. Graphs for Figs 4, 6 and 7c were prepared in Microsoft Excel 2003, for Fig. 7a,b – in SigmaPlot 8. Headings, legends and axes legends were then unified using Adobe Photoshop CS5.

## Electronic supplementary material

Supplementary Figures and Tables
