# Supplementary material for: The genes of the sulphoquinovose catabolism in Escherichia coli are also associated with a previously unknown pathway of lactose degradation
Source: Sci Rep. 2018 Feb 16;8:3177. doi: 10.1038/s41598-018-21534-3 (PMC5816610; doi:10.1038/s41598-018-21534-3)
Supplement: Supplementary file 1 — Supplementary Figures and Tables [file 41598_2018_21534_MOESM1_ESM.pdf]

The genes of the sulphoquinovose catabolism in *Escherichia coli* are also associated with a previously unknown pathway of lactose degradation

Anna Kaznadzey[1], Pavel Shelyakin[1,2], Evgeniya Belousova[3] Aleksandra Eremina[4], Uliana Shvyreva[5], Darya Bykova[3], Vera Emelianenko[3], Anastasiya Korosteleva[6], Maria Tutukina [5,7,*] Mikhail S Gelfand[1,3,7,8]

1 – A. A. Kharkevich Institute for Information Transmission Problems, RAS, Bolshoy Karetny per. 19, Moscow, 127051, Russia
2 – N. I. Vavilov Institute of General Genetics, RAS, ul. Gubkina 3, Moscow, 119991, Russia
3 – M. V. Lomonosov Moscow State University, Vorobievy Gory 1-73, Moscow, 119991, Russia
4 – The University of Edinburgh, Alexander Crum Brown Rd, Edinburgh, Scotland, EH9 3FF, UK
5 – Institute of Cell Biophysics, RAS, Institutskaya 3, Pushchino, 142290, Russia
6 – Novosibirsk State University, ul. Pirogova 2, Novosibirsk, 630090, Russia
7 – Center for Data-Intensive Biomedicine and Biotechnology, Skolkovo Institute of Science and Technology, Moscow, 143028, Russia
8 – Faculty of Computer Science, Higher School of Economics, Kochnovsky pr. 3, Moscow, 125319, Russia

* Corresponding author. E-mail: m.tutukina@skoltech.ru

Table S1. *Escherichia coli* strains and plasmids used in this study

Strains	Description	Reference
K-12 MG1655	Wild type F- lambda- *ilvG*- *rfb*-50 *rph*-1	47
M182	*Δlac* derivative of K-12 MG1655	40
MG1655Δ*yihW*	*ΔyihW* derivative of K-12 MG1655	This work
MG1655Δ*crp*	*Δcrp* derivative of K-12 MG1655	48
M182Δ*yihW*	*ΔyihW* derivative of M182	This work
M182Δ*crp*	*Δcrp* derivative of M182	49
BL21*(DE3)	F- *ompT hsdSB* (rB-mB-) *gal* dcm *rne*131 (DE3)	50
Plasmids		
pET_CRP	Vector based on pET28b (Invitrogen) overproducing the CRP protein, Kan^R	This work

Table S2. Oligonucleotide primers used in this study.

Primer name	Primer sequence (5'-3')
yihU/V_F	CGTTCACATCAAAGACGCGA
yihU/V_R	GTCGGTAACCCTTCCACGTA
yihV/W_F	TCAACCGGCCTTCAAAGTTG
yihV/W_R	GCGATCAGCATGAGGAGTTG
yihS/R_F	AGCTGGATGCGGACAATAAG
yihS/R_R	GGCATCTCTTCGGGTTTGTG
yihW_RT	CCGTATTAACGACGCTGGAA
yihW_PCR	GCCGAGCGTGGGTATATGAA
yihV_RT	TCATCACCTACGCGACCAAT
yihV_PCR	TTCGTGTTGCTTGTGTAGGT
yihU_RT	GGAGTCGCACCTTTGTCTAC
yihU_PCR	CGCGTTTATCGGTTTAGGAC
yihT_RT	ATTGTTGATCTACCAGAATCG
yihT_PCR	CGAAGCCATGCGCATGATGT
yihS_RT	CCGTGATCAACCAACGAGTA
yihS_PCR	GGTTTTGGCTGGTTAGGCAA
hns_RT	ATTTAACGGCAGCAAGGCTATT
hns_PCR	GAAGTTGAAGAGCGCACTCG
hns_Bgl_263	AGGGAGATCTCGTAAACACAACTA
hns_Xba	GTTGTCTAGAATTTTAAGTGCTTCG
CRP_NdeI	ACCGCATATGGTGCTTGGCAAACCGCAA
CRP_Bpu1102	CCACGCTGAGCGGATTAACGAGTGCCGTA

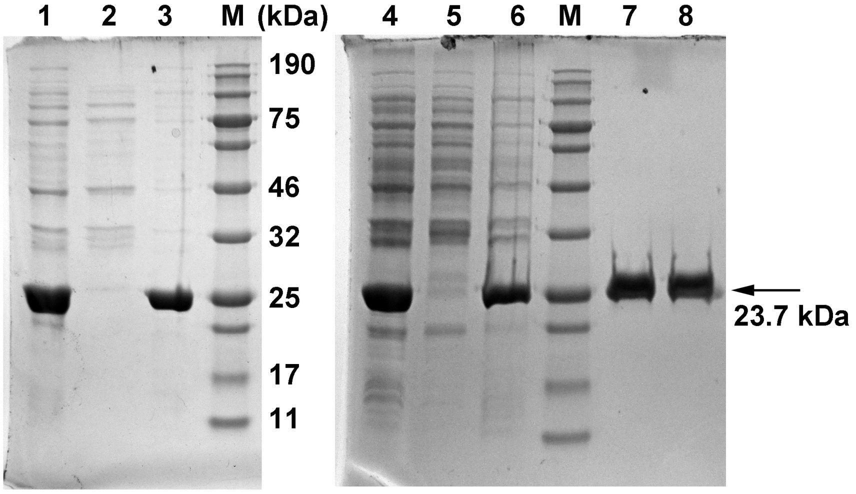

Fig. S1. CRP induction and purification steps. 1 and 4 – total lysate after 16h induction with 20μM IPTG; 2 – soluble fraction; 3 – insoluble fraction; 5 – flow-through after loading of the final lysate (shown on lane 6) onto a column with cAMP-agarose; 6 – cleared cell lysate after treatment with BugBuster reagent (Novagen) followed by sonication; 7 and 8 – pure CRP eluted with 5mM cAMP. Blue prestained protein ladder (11-190 kDa, New England Biolabs) was used as a standard.

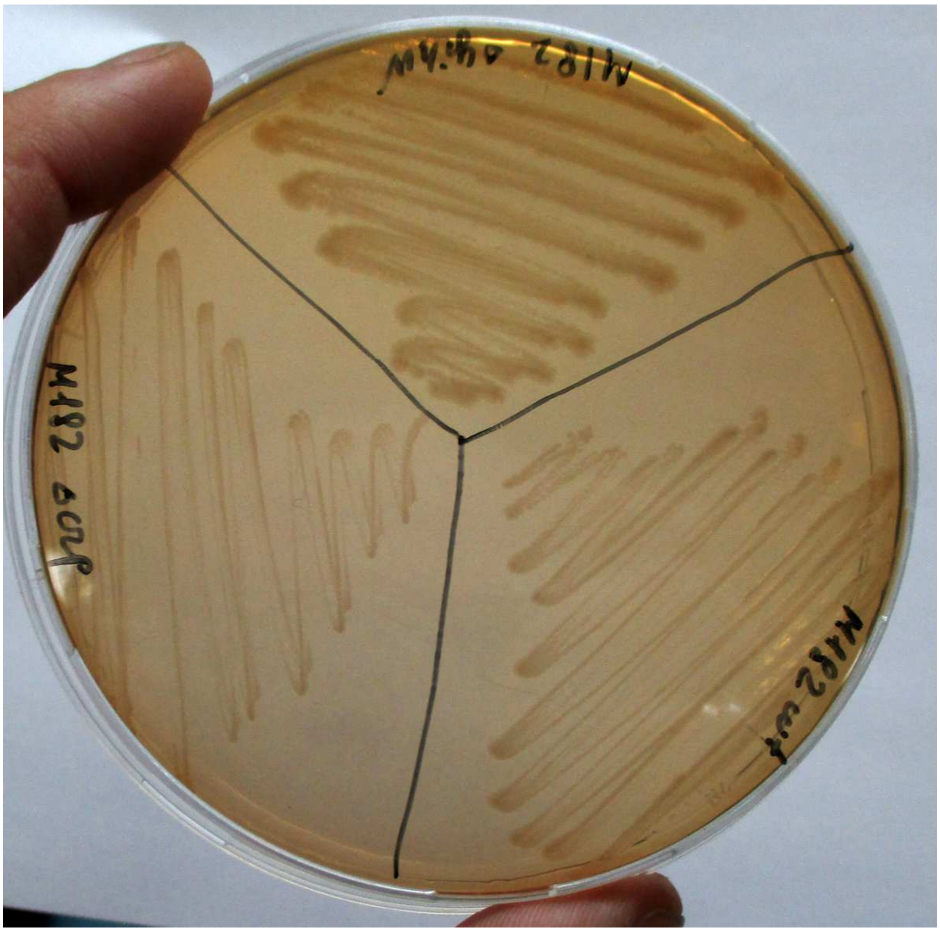

Fig. S2 Growth of the *Escherichia coli* M182 wild type strain, and its Δ*yihW* and Δ*crp* derivatives on MacConkey agar.

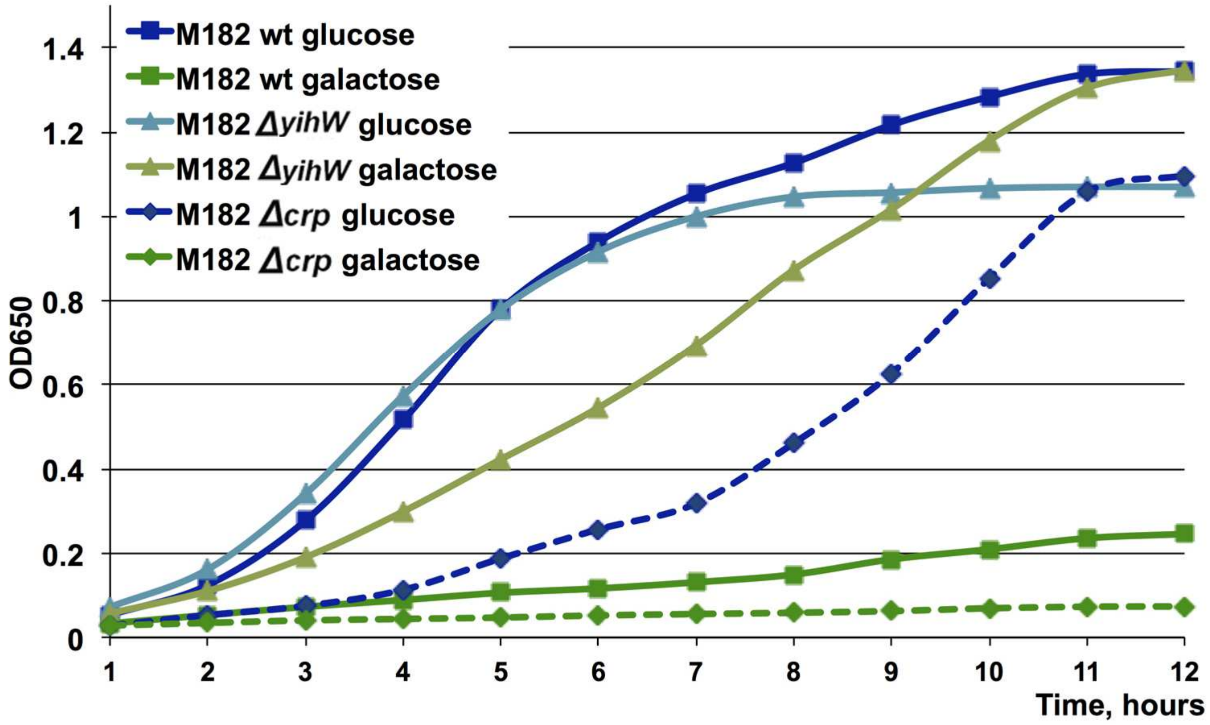

Fig. S3. Effects of *yihW* and *crp* deletions on growth in the presence of 0.2 % glucose or galactose. Squares: the parent strain; triangles: the *yihW* mutant. Dashed lines correspond to cultures with deleted *crp*.
